# Supplementary material for: The interactome of intact mitochondria by cross-linking mass spectrometry provides evidence for coexisting respiratory supercomplexes
Source: Mol Cell Proteomics. 2017 Dec 8;17(2):216–32. doi: 10.1074/mcp.RA117.000470 (PMC5795388; doi:10.1074/mcp.RA117.000470)
Supplement: Supplemental Data [file supp_RA117.000470_133922_0_supp_23978_fzffwf.zip › spectra_annotation/mito_DR_spectra_annotation/135-1-11-1-11-1.pdf]

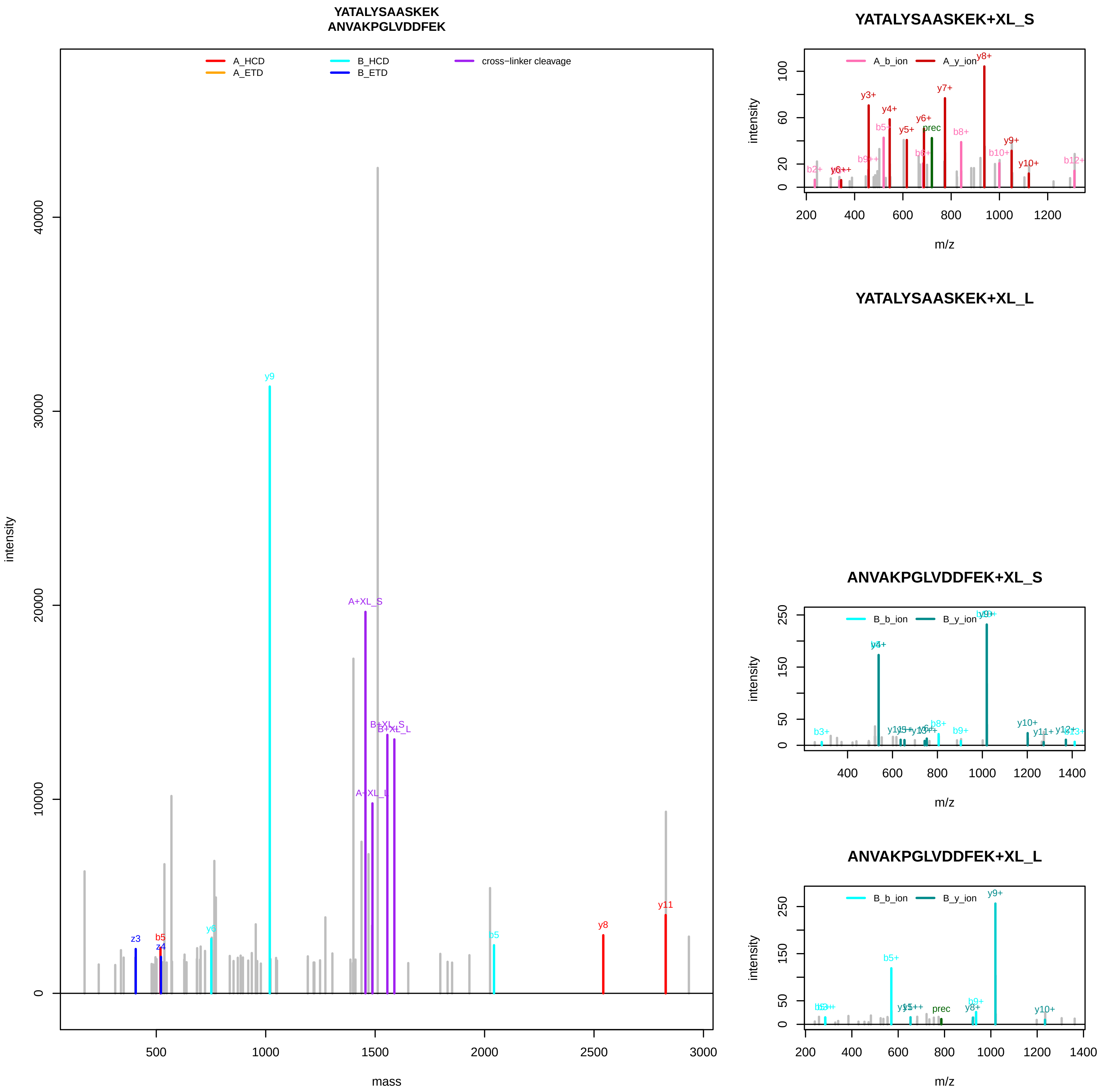
YATALYSAASKEK
ANVAKPGLVDDFEK
A_HCD
A_ETD
B_HCD
B_ETD
cross−linker cleavage
intensity
mass
y9
A+XL_S
B+XL_S
B+XL_L
A+XL_L
z3
b5
z4
y6
b5
y8
y11
YATALYSAASKEK+XL_S
A_b_ion
A_y_ion
y8+
y7+
y3+
y4+
y6+
y5+
prec
b8+
y9+
b10+
y10+
b12+
b2+
m/z
YATALYSAASKEK+XL_L
ANVAKPGLVDDFEK+XL_S
B_b_ion
B_y_ion
y4+
y10+
b3+
b8+
b9+
y11+
y12+
ANVAKPGLVDDFEK+XL_L
y9+
b5+
prec
b9+
y8+
y10+
